# Is IP-10 a Better Biomarker for Active and Latent Tuberculosis in Children than IFNγ?

**DOI:** 10.1371/journal.pone.0003901

**Published:** 2008-12-09

**Authors:** Elizabeth Whittaker, Andrea Gordon, Beate Kampmann

**Affiliations:** 1 Department of Paediatrics, Imperial College London, London, United Kingdom; 2 Wellcome Centre for Clinical Tropical Medicine, Imperial College London, London, United Kingdom; University of British Columbia, Canada

## Abstract

**Background:**

The blood based interferon-gamma release assays (IGRA) for the diagnosis of tuberculosis do not discriminate between active TB disease and latent TB infection (LTBI). The search for distinguishing biomarkers therefore continues, as the accurate diagnosis of tuberculosis is particularly challenging in children. IFN-γ-inducible protein 10 (IP-10/CXCL10) has recently been evaluated as a marker for active TB in adults with promising results.

**Aim:**

To investigate this new biomarker for active TB and LTBI in paediatrics.

**Method:**

We measured IP-10 levels using ELISA in supernatants of whole blood samples stimulated with TB-specific-antigens and negative control antigen.

**Results:**

IP-10 is produced in high levels following mycobacterial antigen stimulation in active TB (n = 17) and LTBI (n = 16) compared to controls (n = 16) and to IFN-γ. The baseline levels of IP-10 are increased in active TB and in LTBI, but there is no significant difference of stimulated levels of IP-10 between active TB and LTBI.

**Conclusions:**

IP-10 is a biomarker for tuberculosis in children. However like IFNγ, IP-10 also does not distinguish between active TB and LTBI.

## Introduction

The diagnosis of tuberculosis (TB) in the paediatric population remains a challenge, as TB can present similarly to many common childhood infections and shares many symptoms with HIV. Diagnosis is often based on contact history, clinical picture, tuberculin skin test (TST) responses and investigations including chest x-ray (CXR) rather than on the microbiological “gold standard” [1]. The paucibacillary nature of TB in childhood limits the microbiological yield and immunodiagnostic methods such as the Mantoux test have been widely used in paediatrics for many years[2]. In recent years new immunodiagnostic tests for tuberculosis have been developed: the commercially available Quantiferon TB Gold In Tube (Cellestis, Carnegie, Australia) and T-SPOT TB (Oxford Immunotec, Abingdon, UK) ELISPOT assays both measure interferon gamma (IFN-γ) release by sensitized T cells after stimulation with peptides of *M. tuberculosis* specific antigens - Early Secretory Antigenic Target (ESAT)-6, Culture Filtrate Protein (CFP)-10 plus TB7.7 in the Quantiferon system.[3] These interferon gamma release assays (IGRA) are less influenced than the TST by factors frequently associated with childhood tuberculosis in developing countries, such as malnutrition and HIV co-infection [4]. BCG vaccine and environmental mycobacterial exposure are also known to influence TST results but not IGRA[5], [6]. However, the IGRA poorly distinguish between active TB and latent TB infection (LTBI) and more recent studies question the sensitivity of these assays, particularly in some groups including younger children and immunocompromised patients. [7], [8], [9].

Recent studies looking at alternative markers for diagnosis of TB have identified a promising chemokine called IFN-γ- inducible protein 10 (IP-10) [10]. Also known as CXCL10, it is a member of the CXC-chemokine family. Chemokines are a class of cytokines with chemotactic properties; they have a role in mediating leukocyte migration and activation. IP-10 is expressed in both lymphocytes and monocytes and is involved in trafficking of Th1 lymphocytes to areas of inflammation where it binds to CXCR3 (a receptor shared by IP-10, Mig and I-TAC)[11]. CXCR3 mRNA is expressed in T cells alone and thus IP-10 differs from other chemokines as it targets lymphocytes specifically and has no activity on neutrophils. In adult studies, high levels of IP-10 have been found in delayed type hypersensitivity reaction to Tuberculin Purified Protein Derivative (PPD)[12], in vivo in lymph node and lung tuberculous granulomas[13], in pleural effusions and plasma of TB infected patients[14], [15] and plasma of TB-HIV co-infected patients diagnosed with immune reconstitution syndrome (IRS)[16]. IP-10 has been suggested as a marker of TB treatment efficacy – serial samples show reductions in levels at 2 months in cured TB patients, with no reduction in non-responders [15]. Levels of IP-10 are higher in those patients with systemic symptoms such as fever and anorexia[16]. Of note IP-10 levels in household contacts of TB infected patients, both adult and children (85% older than 5 years), were higher than controls, suggesting a potential value for latent TB infection (LTBI) diagnosis [15], [17]. In view of these findings in predominantly adult studies, we investigated whether IP-10 could be a new diagnostic biomarker for TB in children, specifically, whether it could help in distinguishing between active TB and LTBI – a particular challenge in the paediatric population.

## Methods

### Subjects

Study approval was obtained from the ethics committees of Imperial Healthcare NHS Trusts and North West London Hospitals Trust.

We recently conducted a research study comparing TST and IGRA in the diagnosis of active TB and LTBI in children in London, using the QFG-IT test and T Spot TB (submitted for publication). In this study, we included all children between 2 months and 16 years of age who were investigated for active TB, as well as referrals for contact tracing after exposure to a known case of active TB. Exclusion criteria were immunocompromise, including immunosuppressive therapy and HIV. Remaining supernatant samples harvested from the QFG tubes and not used for the determination of IFNγ were used in this additional investigation. For the purpose of the IP-10 study, only children with a TST>15 and positive QFG-IT were selected for the cohorts of active and latent TB.

Written consent for the IGRA study, including consent to use remaining samples, was obtained from all study participants.

#### Definition of active TB

The diagnosis of active TB was based on the presence of acid-fast bacilli in either gastric washings/induced sputum or other microbiological samples, and confirmed by growth in culture. Only children with culture proven TB were included in this study.

#### Definition of latent TB infection (LTBI)

For the purpose of this study, only children with a TST>15 and positive QFG-IT were selected for the cohort of LTBI. None of the children had signs and symptoms of active TB and a normal chest X- ray.

#### Non-TB Control group

16 children with non-TB disease were included as controls. They were recruited from both the outpatient department and the general paediatric ward and had a variety of alternative diagnoses, such as fractures, urinary tract infections, pneumonia, allergy and nephrotic syndrome. Careful history excluded TB contact. Both IGRA were performed on all these children and were negative in all 16.

#### Whole Blood Stimulation

Briefly, 1 ml of blood was drawn directly into each vaccutainer tube provided as part of the QFG-IT system (Cellestis, Carnegie, Australia). These tubes are already precoated with saline (negative control), peptides of ESAT-6, CFP10 and antigen TB 7.7 (referred to as antigens/Ag for the remainder of the paper) - and PHA (positive mitogen control). The tubes were mixed and incubated for 20–24 hr at 37°C, when plasma was harvested and frozen until further analysis. This plasma provided the unstimulated and antigen (Ag) stimulated supernatant samples.

#### IP-10 quantification

IP-10 supernatant levels were measured using a sandwich ELISA. Briefly, ELISA plates were coated overnight with a monoclonal antibody for Human CXCL10/IP-10 (R&D Systems), blocked with a 2% bovine serum albumin solution, extensively washed and test samples or recombinant human CXCL10/IP-10 (R&D Systems) as standards were added and incubated at room temperature for one hour. After washings, a biotinylated polyclonal secondary antibody to human CXCL10/IP-10 as (R&D Systems) was added and incubated for 1 hour at room temperature. After washing, streptavidin conjugated HRP (Biosource), was added for 15 minutes at room temperature, followed by TMB. The reaction was stopped with 2 m H2SO4 and absorbance at 450 nm was measured in an ELISA reader. The lower end of sensitivity was 50 pg/ml for this assay. The upper limit of IP-10 for the assay was 40,000 pg/ml and readings greater than this were set at 40,000 pg/ml for the purpose of analysis.

#### Calculation of Stimulation Index

For comparison purposes, a stimulation index was calculated (ie the cytokine concentration in plasma of antigen- stimulated whole blood divided by the cytokine concentration of unstimulated whole blood). A stimulation Index greater than 1.25 indicates that there was a significant response to Ag stimulation [10].

#### Statistical Analysis

Significant differences between cytokine levels were determined by the Kruskal-Wallis test. If a significant difference was found, the Mann-Whitney U-test was used to calculate differences between each pair of groups. Paired data was compared by the (two-tailed) Wilcoxon signed rank test. P values were two-sided and considered significant if <0.05. All calculations were performed using GraphPad Prism computer program (GraphPad Software Inc., San Diego, CA, USA).Data shown in the figures and tables are given as medians.

## Results

The median age of the study group was 10.15 yrs (range 0.4–16.8 yrs). The 17 children with active TB ranged from 5 to 16 years with a median age of 11.3, of whom 9 had received BCG vaccination. The 16 children with LTBI had a median age of 10.5 years (range: 5–15.5 yrs), and 12 children were BCG vaccinated. The median age of the control group was 5.9 years (range: 0.4–16.8 yrs) and 8 children had received BCG-vaccine.

### IP-10 measurements

**Figure 1:**
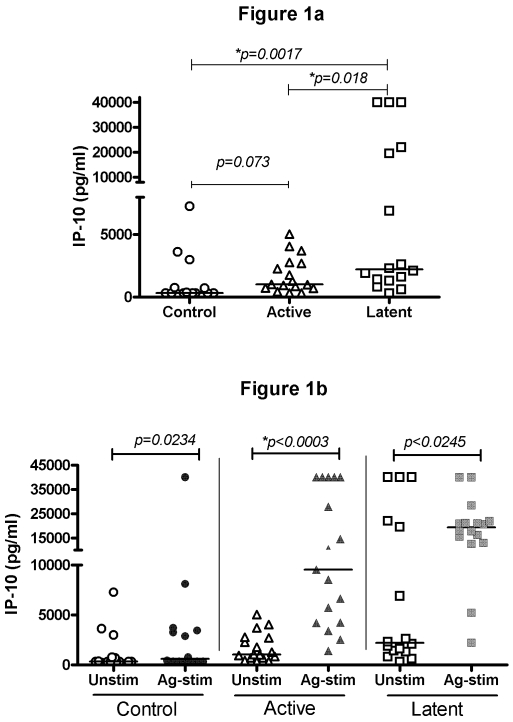
Concentration of IP-10 (pg/ml) in supernatants from: a) whole blood samples incubated with negative control antigen (background); b.) whole blood samples incubated with TB-specific antigens and negative control antigen, of children with active TB (n = 17), LTBI (n = 16) and a control group (n = 16). Horizontal lines indicate median values. Controls are represented by ○, active TB by ▵, and LTBI by □. Open symbols represent unstimulated, closed symbols represent antigen-stimulated samples.

The unstimulated or background levels of IP-10 were increased in the active TB group (median 1019 pg/ml, range: 320–5047 pg/ml) compared to control (median 320 pg/ml, range: 320–7275 pg/ml, p = 0.073). However the level of IP-10 in the LTBI group (median 2200 pg/ml, range: 320–40,000 pg/ml) were significantly greater than either active TB (p = 0.018) or control, (median 320 pg/ml, p = 0.0017). (Figure 1a)

The TB specific antigen stimulated samples demonstrated highly significant differences between active TB (median 9544 pg/ml, range: 1386–40,000 pg/ml) and LTBI (median 19350 pg/ml, range: 2200–40,000 pg/ml, p = 0.004) compared with the control group (median 536 pg/ml, range: 320–40,000 pg/ml, p = 0.0085). (Figure 1b) When comparing the active TB and LTBI cases, there was a greater increase in the antigen-stimulated samples from the patients with LTBI (median 19350 pg/ml) than in the patients with active TB (median 9544 pg/ml); however, this was not significant (p = 0.85).

In the patients with active TB, levels of IP-10 were found to be significantly increased in the TB specific antigen stimulated samples (median 9544 pg/ml, range: 2535–40,000 pg/ml) as compared to the unstimulated or background samples (median 1019 pg/ml, 433–4308 pg/ml; p<0.0003) (Figure 1a).

A significant increase in IP-10 levels following antigen stimulation was also detected in the samples from patients with LTBI (median 19350 pg/ml, 2200–40,000 pg/ml, p<0.0245, Figure 1b), however, less significant than in active TB, most likely due to the higher baseline levels.

This antigen- stimulated increase in IP-10 was also noted in the control group, (median increased from 320 pg/ml to 536 pg/ml; p = 0.0234) but significantly lower than in active TB. This is largely due to one patient having a very high level (40,000 pg/ml) following antigen stimulation.

### Levels of IP-10 compared with IFN-γ

**Figure 2:**
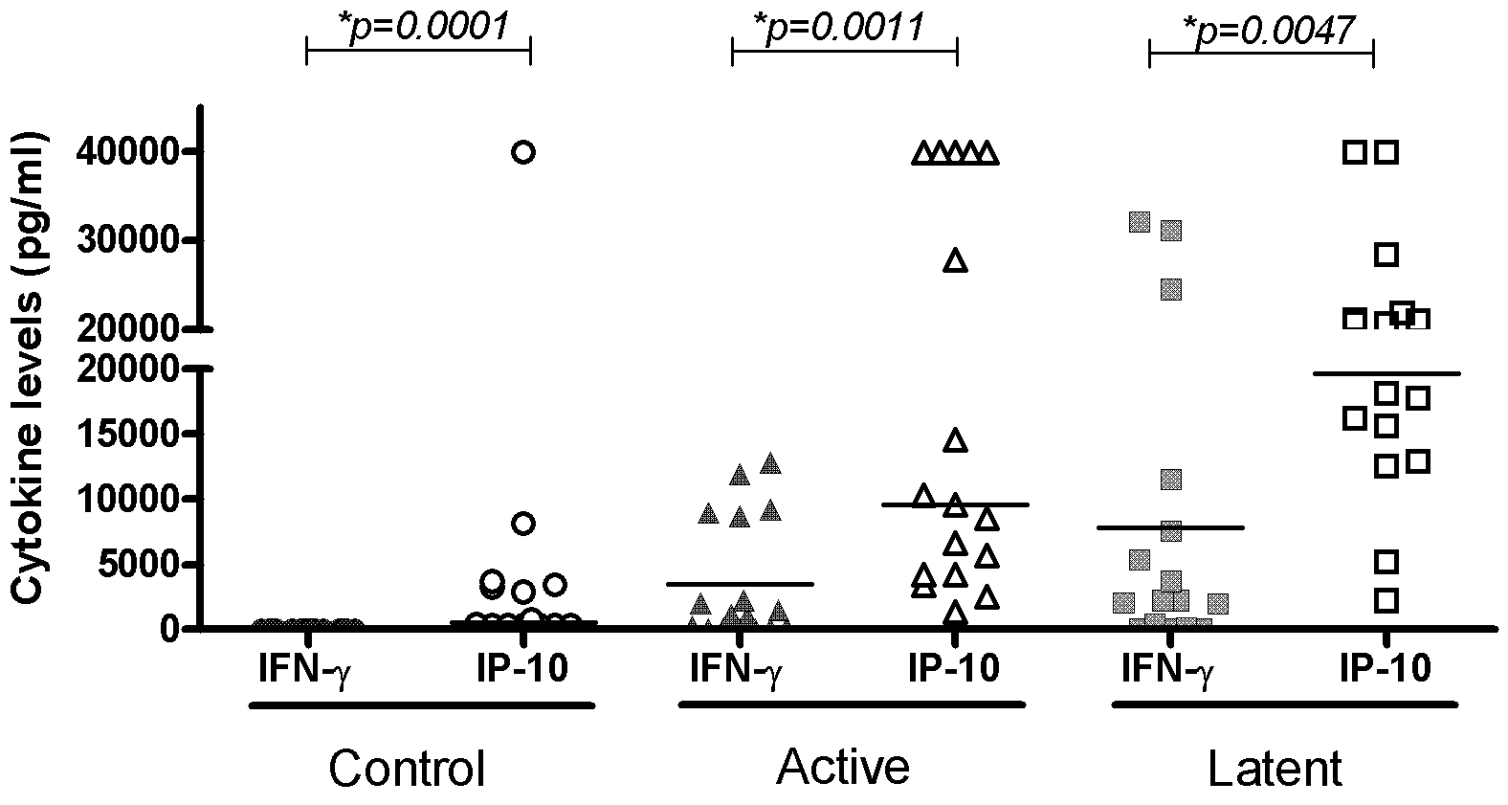
IFN-γ and IP-10 levels (pg/ml) in Antigen stimulated samples of patients with active and latent TB and a control group. Horizontal lines indicate median values. Controls are represented by ○, active TB by ▵, and LTBI by □. Open symbols represent IP-10, closed symbols represent IFNγ samples.

The levels of IP-10 were compared to the previously measured levels of IFN-γ in the same patients using the QFG-ELISA system. In all three groups, there were significantly higher levels of IP-10 than of IFN-γ following antigen stimulation (Control - IFNγ median 7.25 pg/ml, range 3.5–46.5 pg/ml; IP-10 median 536 pg/ml, range: 320–40,000 pg/ml:p = 0.0001; LTBI - IFNγ median 2205 pg/ml, range 60–31084 pg/ml; IP-10 median 19350 pg/ml, range 2,200–40,000 pg/ml, p = 0.0047: Active TB - median IFNγ 1124.5 pg/ml, range 10–11928.5 pg/ml; IP-10 median 9544 pg/ml, range 1386–40,000 pg/ml, p = 0.0011: (Figure 2). In the group with LTBI, the levels of both IP-10 and IFN-γ were higher than in patients with active TB (IP-10 p = 0.85; IFNγ p = 0.05).

### Stimulation Index

**Figure 3:**
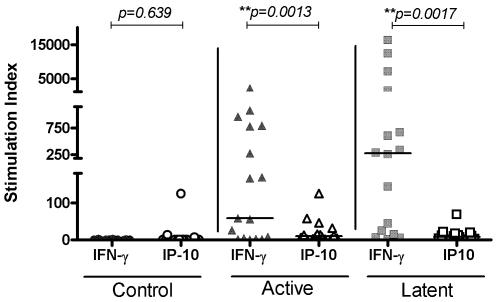
Stimulation index of IP-10 and IFN-γ in active TB, LTBI and a control group. Horizontal lines indicate medians. Controls are represented by ○, active TB by ▵, and LTBI by □. Open symbols represent IP-10, closed symbols represent IFNγ samples.

Figure 3 shows that the stimulation index (SI) of IP-10 in both active (median SI: 9.4, range: 1–125) and latent TB groups (median SI: 8.8, range: 0.32–68.7) are greater than in the control group (median SI: 1.7, range 0–125). This is significant in the active cases (p = 0.04), but not the LTBI cases (p = 0.23). There is only a slight difference in stimulation index between active and latent TB (p = 0.09).

The stimulation index was also used to compare IFN-γ and IP-10 in all three groups of patients (Figure 3). There was no difference between the IP-10 and IFNγ stimulation indices in the control groups, but IFN-γ has a significantly higher stimulation index than IP-10 in both the active TB (median SI IFNγ: 112.5, range: 1–2131: median SI IP-10: 9.4, range: 1–125; p<0.0013) and latent TB groups (median SI IFNγ: 271, range: 1.5–16331, median SI IP-10: 8.8, range: 0.32–68.7; p<0.0017).

## Discussion

This is the first published study comparing the use of IP-10 as a biomarker for active and latent tuberculosis in children. Although we have demonstrated that IP-10 is a marker for tuberculosis – active and latent - in children, we did not find it to be discriminative between the two disease states.

Chemokines are believed to play a role in the pathogenesis of tuberculosis, specifically in granuloma formation[18]. In particular, raised levels of IP-10 are found in adult studies of patients with active TB[10]. Elevated levels have also been noted in household contacts of patients with active TB[15]. Many of these studies were measuring the unstimulated plasma level of IP-10 in confirmed cases of TB[19]. In our study we noted a high background level of IP-10, confirming this previously published work. It is noteworthy that the background level of IP-10 is significantly greater in LTBI than active TB, possibly due to a chronic state of inflammation as the immune system attempts to control TB infection. Alternatively, an immunosuppressive effect of active TB, modifying cytokine responses might also explain the differences seen. It is unclear at this stage whether the background levels of IP-10 could carry a prognostic value for the development of active TB from latent TB.

We showed that IP-10 is produced in high levels following MTB antigen-specific stimulation of whole blood in both active TB and LTBI cases, demonstrating its potential as a biomarker for MTB in children. Ruhwald et al reported similar findings in adults with culture positive tuberculosis[10], and in children with presumed latent TB infection[17], but there are no published data for active TB in a paediatric population. The paediatric study by Ruhwald included 97 household contacts under 15 years of age (85%>5 yrs) and compared IP-10 levels in child contacts of smear positive adults with those in both smear negative contacts and a control group. A significant increase in IP-10 was found in the smear positive contact group. However, these children did not have active TB, as this study was essentially a household contact investigation. There was good correlation between the QFG-IT and IP-10 results in all groups in this study (93/120, 78%, κ = 0.64) [17]. Age ranges in both the Ruhwald study and our own study were similar and produced comparable results for LTBI.

In our study, IP-10 was produced in significantly higher levels than IFN-γ, in response to MTB antigens. IFN-γ producing T-cells are known to induce IP-10 production in primary exposure to TB. In the mouse model, IP-10 is induced by IL-23 and IL-17 producing T-cells in a recall response to MTB infection in the lung, independent of IFN-γ production [20]. IL-23/IL-17 secreting T-cells are known to establish IFN-γ producing T-cells following initiation by TGF-β and IL-6[20]. These regulatory cytokines are likely to be key players in granuloma stabilisation and therefore latent TB; induction by both IFN-γ-producing and IL-17-producing T-cells may explain why the levels of IP-10 are greater in latent TB than active TB.

The stimulation index indicates a positive response to the specific MTB antigens as opposed to generalised activation of cytokine- producing cells. The stimulation index of IP-10 was notably lower than that of IFN-γ, despite the higher levels of cytokines produced. This is due to the higher background level of IP-10 in unstimulated samples, particularly noticeable in the latent TB cases as discussed above.

IP-10 levels are increased in patients with inflammatory states such as autoimmune disease (systemic lupus erythematosus, thyroid disease), acute coronary syndrome, cerebral malaria and allergy[21], [22], [23], [24]. In the control group in our study, one patient in particular was found to have very high levels of IP-10. It is probable that IP-10 is mainly a marker of inflammation and therefore too non-specific to be a stand-alone biomarker for TB infection. However it may still have a role as part of a panel of diagnostic markers.

IFN-γ is the key cytokine produced in response to TB antigens following TB exposure causing either active or latent TB and this observation forms the basis of the IGRA. However, IGRA do not distinguish between active TB and LTBI. It is interesting to note that although we found a higher IFNγ stimulation index in LTBI than in active TB in our study, unfortunately the difference is not significant and therefore not useful as a diagnostic test. A difference in IFNγ levels in active TB and LTBI has been noted before and some groups suggest a role for CD4^+^CD25^high^FoxP3^+^ T-cells in suppressing IFN-γ responses to a variety of TB antigens in active TB [25]. An alternative hypothesis is that IFN-γ producing CD4^+^ T cells are sequestered at the tissue site of infection, rather than in the blood, causing a paradoxically low level of IFN-γ production to antigen stimulation. This may also explain the lower levels of IP-10 in active TB compared to latent TB.

The main limitation of this study is the small sample size, which could potentially result in a type II error in the statistical analysis. However, we think, that this is unlikely, as all of the children in each group were very well characterised, thus the results represent a trend that provides direction for further studies. The children with active and LTBI all had TST>15 and positive IGRA tests, hence strong evidence of mycobacterial infection. We deliberately selected these well-characterised phenotypes in order to directly compare the stimulation of IP-10 with IFN-γ production. This could have led to bias in the IP-10 results as its production can be induced by IFN-γ. It would be interesting to compare IP-10 and IFNγ levels also in those children with clinically diagnosed TB and negative IGRA.

Although IP-10 does not appear to be sensitive or specific enough to supersede IGRA as tools for TB diagnosis, or to distinguish between active TB and LTBI, a number of conclusions can be drawn from our study: the supernatants of the Quantiferon Gold-In-Tube assays are a useful and robust resource for investigation of further proposed biomarkers, and we are currently investigating other cytokines as potential biomarkers to distinguish between active TB and LTBI using this sample bank. The levels of IP-10 in unstimulated blood of LTBI patients may have diagnostic and prognostic value for further investigation, and higher levels may indicate a potentially protective mechanism for the development of LTBI rather than active TB. Of course, only prospective evaluation of such a biomarker could confirm this hypothesis.
